# The complete mitochondrial genome of the Cape honey bee, *Apis mellifera capensis* Esch. (Insecta: hymenoptera: apidae)

**DOI:** 10.1080/23802359.2016.1241682

**Published:** 2016-11-12

**Authors:** Amin Eimanifar, Rebecca T. Kimball, Edward L. Braun, James D. Ellis

**Affiliations:** a Honey Bee Research and Extension Laboratory, Department of Entomology and Nematology, University of Florida, Gainesville, FL, USA;; b Department of Biology, University of Florida, Gainesville, FL, USA

**Keywords:** Cape honey bee, mitogenome, next-generation sequencing, *Apis mellifera capensis*, South Africa, social parasite

## Abstract

We characterized the complete mitogenome sequence of the Cape honey bee, *Apis mellifera capensis*, from South Africa. The circle genome is 16,470 bp in length, with the base composition of 43.2% A, 9.6% C, 5.6% G, and 41.5% T. The assembled mitogenome has 13 protein-coding genes (PCGs), 22 transfer RNAs, two ribosomal RNA genes, and one control region. All protein-coding genes are initiated by ATT, ATC, ATG or ATA codons and are terminated by the typical stop codon TAA. The heavy strand encodes four protein-coding genes, eight tRNAs, and two rRNAs. The light strand encodes nine protein-coding genes and 14 tRNAs. The complete mitogenome sequence of *A.m. capensis* is identical to the gene arrangement found in other *A. mellifera* mitogenomes and it provides essential and important DNA molecular data for further phylogenetic and evolutionary analysis of members of the genus *Apis*.

Africa is the home to at least 10 indigenous *Apis mellifera* L. (western honey bee) subspecies that are distributed across the continent with substantial geographical, climatic, and ecological variability (Gupta et al. 2014). *Apis mellifera scutellata* and *Apis mellifera capensis* are two important subspecies inhabiting South Africa (Hepburn & Radloff 1998). *Apis mellifera capensis*, the Cape honey bee, is a facultative social parasite (Cape bee workers invade other non-Cape bee colonies, becoming the resident reproductive), reproducing thelytokously (unfertilized eggs become diploid females), and is characterized by a unique set of genetic, behavioural, and physiological traits possessed by the worker bees (Onions 1912; Hepburn & Crewe 1991; Neumann & Moritz 2002).

In this study, we report the complete mitogenome (mitochondrial genome) of an *A.m. capensis* worker (Accession no. KX870183) collected from an apiary close to Knysna, a city that lies in the natural distribution of *A.m. capensis* in South Africa (34°05′S–22°99′E (Hepburn & Radloff 1998). The identity of the worker bee was confirmed using classic morphometrics as per Hepburn and Radloff (1998). Total genomic DNA was isolated from a worker honey bee as per Hunt & Page (1995) using cetyltrimethylammonium bromide (CTAB), followed by phenol:chloroform:isoamyl alcohol (25:24:1). The genomic DNA was quantified using a Qubit^®^ 3.0 Fluorometer (Thermo Scientific Inc., Waltham, MA, USA). Genomic libraries were constructed and genome skimming (Straub et al. 2012) was performed using pair-end sequencing (2 × 100 bp) on the Illumina HiSeq 2000 (San Diego, California, USA) sequencing platform.

The resulting FASTQ reads were trimmed and mapped to the reference mitogenome of *A.m. ligustica* (L06178.1, the Italian honey bee) using Geneious R9.1 (Kearse et al. 2012). We used 10 iterations of custom-settings to ensure high-stringency mapping. In regions with ambiguities, we selected short consensus segments (>100 bp) and remapped these with original reads to elongate them without bias of the original reference sequence. We combined these assemblies using 2–3 iterations to achieve a final sequence that was examined by eye. To confirm, a final mapping of the reads was done to the final sequence to ensure there were no errors.

The mitochondrial genome of *A.m. capensis* constitutes a DNA circular closed loop that is 16,470 bp in length. The *A.m. capensis* mitogenome was similar in content and organization to that of *A.m. ligustica*. It contained 13 protein-coding genes, 22 putative tRNAs, two rRNAs, and an AT-rich control region. There was a strong A + T bias (84.7% of the genome). The heavy strand encodes four protein-coding genes, eight tRNAs, and two rRNAs. The light strand encodes nine protein-coding genes and 14 tRNAs. Six protein-coding genes of the *A.m. capensis* mitochondrial genome started with ATT, four with ATG, two with ATA, and one with ATC. The stop codon of each of these protein-coding genes was TAA.

The mitogenome of *A.m. capensis* shared 96% genetic identity with that of *A.m. ligustica*, with 274 nucleotide substitutions and 338 indel differences. There were 32 amino acid differences, occurring in all genes except ND4L. The starting position of ND4 differs among *Apis* mitogenomes. Based on the *A.m. ligustica* mitogenome, which has the longest ND4 annotated for an *Apis mellifera* mitogenome to date (411 amino acids, relative to 409 to 398 in other taxa presented in the Figure 1), the likely homologous start position in *A.m. capensis* resulted in a stop codon at the eighth amino acid position. However, the starting position for ND4 based on annotations of *A.m. scutellata* and *A.m. syriaca*, which exclude these initial sites, did not lead to a stop codon on the translated ND4 sequence of *A.m. capensis*. Thus, we assumed this was the appropriate start codon.

**Figure 1.**
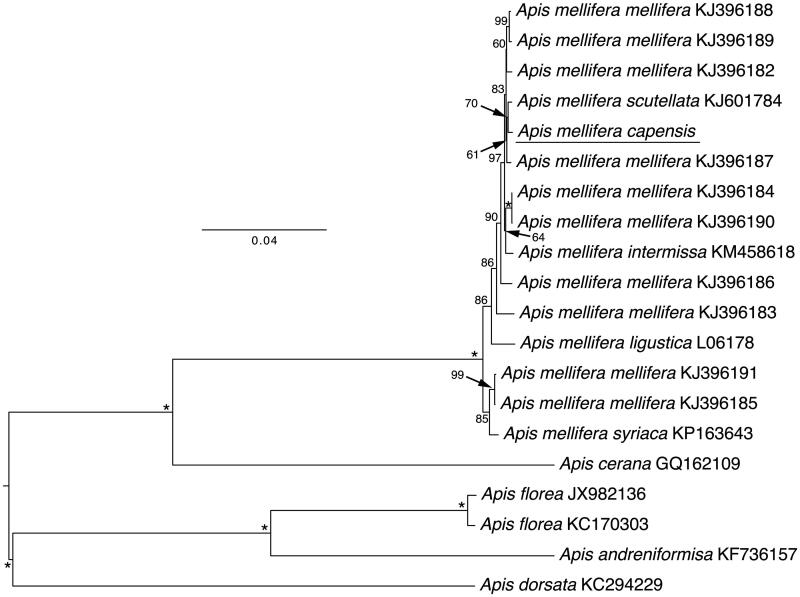
Phylogenetic relationships within the genus *Apis* based on maximum likelihood approach of the mitochondrial genome nucleotide sequence of the 13 protein-coding genes. The bootstrap values are given in numbers on the left of each node. The letter/number combination on the right side of each node indicates the GenBank accession numbers.

There was 99% identity of the *A.m. capensis* mitogenome with that of *A.m. scutellata* (KJ601784), a honey bee subspecies which is distributed in South Africa borders with that of *A.m. capensis*. The differences were due to 44 nucleotide substitutions and 95 sites with indels which were all located in non-coding regions. There were three amino acid differences between the *A.m. capensis* and *A.m. scutellata* mitogenomes, one each in ND2, CO3, and ND5. Furthermore, *A.m. capensis* has an additional amino acid in the ND4 gene that is absent in *A.m. scutellata* (but is present in the other mitogenomes). In total, there were four amino acid differences between *A.m. capensis* and *A.m. scutellata* (3 substitutions and one indel).

We also identified a pseudogene of ∼2000 bp (the exact boundaries were difficult to establish due to assembly issues). This covered ATP6 through ND5, with two large (> 500 bp) deletions, as well as many smaller insertions and deletions relative to the reference genome. Overall identification for the regions covered was over 78%, with high identity in some regions.

A phylogenetic analysis was constructed using the 13 mitochondrial protein-coding genes and two rRNAs with inclusion of 19 published mitogenomes from other species of *Apis* and subspecies of *A. mellifera*. To identify the phylogenetic position of *A.m. capensis*, we used RAxML 7.2.8 (Stamatakis 2006) and the GTRGAMMA model to construct a maximum-likelihood tree; we estimated the ML tree using 10 random additions, and assessed support with 1000 bootstrap replicates (Figure 1). We used midpoint rooting. The tree confirms that *A.m. capensis* is within the *Apis mellifera* clade and is supported as sister taxa to *A.m. scutellata* with 70% support (Figure 1). *Apis mellifera capensis* clusters inside *A.m. mellifera* subspecies, which do not form a monophyletic clade with the mitochondrial genome. The genetic distance between the *A.m. capensis* and *A.m. scutellata* mitochondrial genomes was 0.002. In contrast, the distance between the *A.m. capensis* genome and all other *A. mellifera* mitochondrial genomes averaged 0.006, consistent with genetic distances between African (0.004) and European and Middle Eastern *A. mellifera* (0.014) (Crozier & Crozier 1993; Gibson & Hunt 2015; Haddad 2015; Hu et al. 2015). In conclusion, the complete *A.m. capensis* mitogenome will provide essential and fundamental information to elucidate population genetics, biogeography and evolutionary history of this bee and related subspecies.
